# Gastrojejunal anastomosis in rats using the magnetic compression technique

**DOI:** 10.1038/s41598-018-30075-8

**Published:** 2018-08-02

**Authors:** Yingfeng An, Yanchao Zhang, Hao Liu, Sijie Ma, Shan Fu, Yi Lv, Xiaopeng Yan

**Affiliations:** 1 The First Affiliated Hospital of Xi’an Jiaotong University, Department of Hepatobiliary Surgery, Xi’an, 710061 China; 2 Regenerative Medicine and Surgery Engineering Research Center of Shaanxi Province, Xi’an, 710061 China; 3 Shaanxi Provincial Centre for Disease Control and Prevention, Xi’an, 710054 China; 4 Xi’an Jiaotong university Health Science Center, Qide College, Xi’an, 710061 China

## Abstract

Rats are suitable animal models in which to study the effects of gastric bypass surgery. However, construction of gastrojejunal anastomosis in the rat is technically demanding and is associated with high rate of postoperative complications. The aim of this study was to explore the feasibility and efficacy of the magnetic compression technique (MCT) in side-to-side gastrojejunal anastomosis in rats. Thirty male rats underwent gastrojejunal anastomosis using one of three techniques: hand-sewn, magnetic compression using cuboid magnets, and magnetic compression using magnetic rings. The mean anastomosis time using the magnetic compression technique was significantly less than that of the hand-sewn technique (3.6 ± 0.96 and 6.50 ± 1.58 vs. 14.40 ± 2.37 minutes,). The survival rate was highest in animals treated with magnetic compression using cuboid magnets (100%), followed by animals treated with magnetic compression using magnetic rings (90%) and then hand sewing (70%). The mean burst pressure did not differ significantly between the magnetic compression and hand-sewn anastomoses. Anastomoses constructed by magnetic compression were smoother and flatter than hand-sewn anastomoses. The results showed that MCT is a simple and feasible method for gastrojejunal anastomosis in the rat.

## Introduction

Surgical treatments for obesity and metabolic diseases have become an important topic of research in recent years. Gastric bypass has been reported to cause long term remission of diabetes in patients with severe obesity and type 2 diabetes^1,2^. However, the mechanisms behind this effect are still not clear. Numerous experimental animal studies have been carried out^3^, and the rat is considered an ideal animal model of gastric bypass surgery for type 2 diabetes^4,5^. However performing gastric bypass in rats requires a long and complicated procedure that requires extensive training. A simple and effective method for gastrojejunal anastomosis in rats is yet to be established.

Magnetic compression anastomosis was first performed by Obora in 1978^6^ and have since evolved into a successful surgical technique applied to a variety of anastomoses including vascular anastomoses^7–10^, choledochojejunostomy^11–15^, esophageal anastomoses^16,17^, and in the treatment of rectovaginal fistula^18,19^. These studies have shown that magnetic compression technology is feasible, safe and effective. This study aimed to introduce a novel magnetic method for construction of gastrojejunal anastomosis in rats. To the author’s knowledge, this is the first study to apply the MCT for gastrojejunal anastomoses in rats.

## Results

### Anastomosis time

The MCT anastomoses were constructed significantly more quickly (MCT-A: 3.60 ± 0.96 min; MCT-B: 6.50 ± 1.58 min) than hand-sewn anastomoses (control group; 14.4 ± 2.37 min; P < 0.001).

### Survival rate and postoperative complications

The survival rate was 100%, 90% and 70% in the MCT-A, MCT-B and the control group, respectively. All the expired rats underwent exploratory laparotomy in order to identify the cause of mortality. In the MCT-B group, one rat died due to anastomotic leakage and two rats died due to anastomotic obstruction. The expired rat in the MCT-B group had anastomotic obstruction due to the blockage of the central tube of the parent magnetic ring by the intestinal contents.

### Expulsion time of magnetic anastomosis rings

The mean time required for the magnets to be expelled from the rat were 7.20 ± 1.69 days (range, 5–10 days) and 9.30 ± 1.49 days (range, 7–12 days) after surgery in the MCT-A group and MCT-B group, respectively.

### Bursting pressure

One month after surgery, the mean bursting strength in MCT-A (251.40 ± 30.18 mmHg) and MCT-B (246.67 ± 26.61 mmHg) did not differ significantly from that in the control group (244.57 ± 35.34 mmHg) (P > 0.05).

### Gross appearance of anastomosis

**Figure 1:**
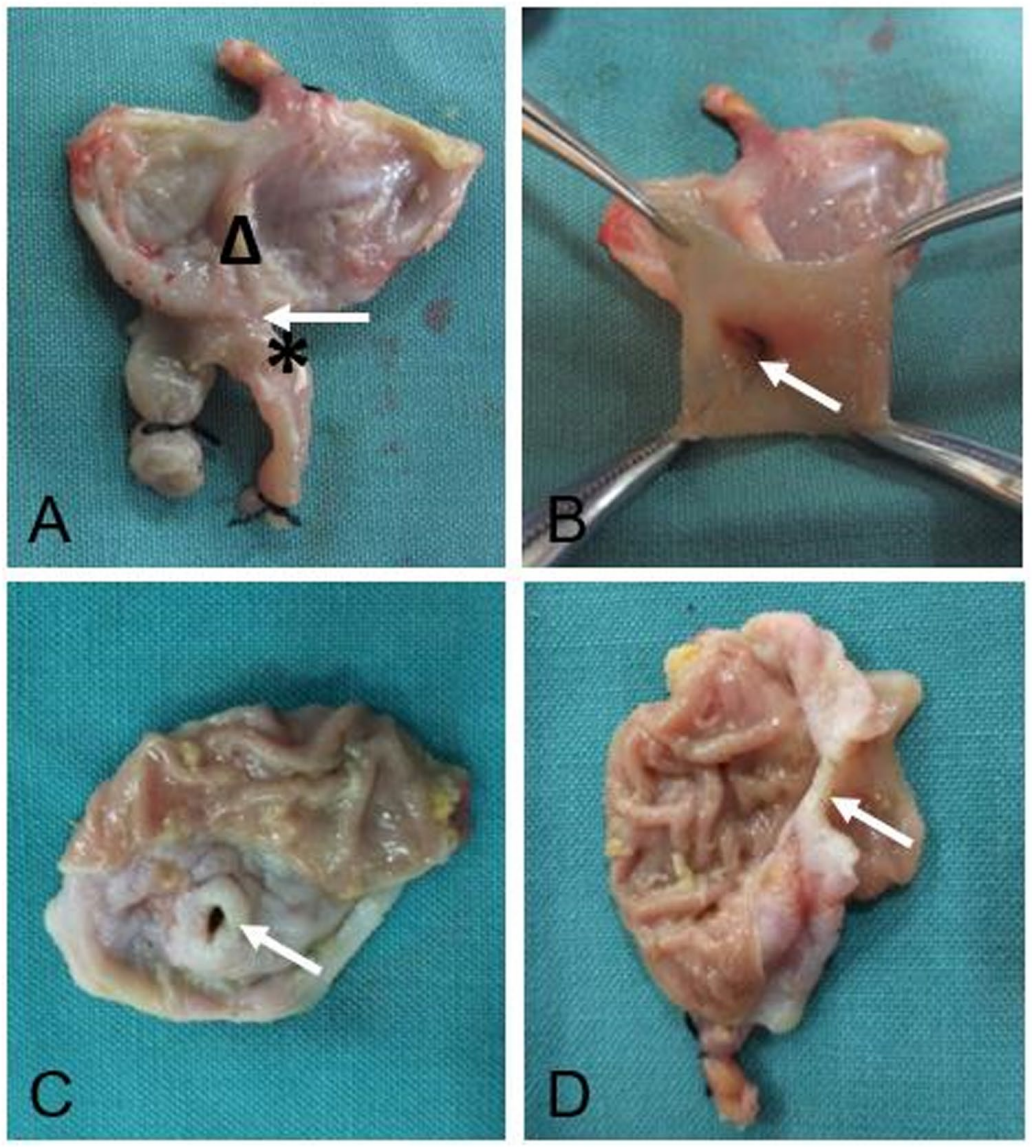
Gross appearance of the anastomosis performed by magnetic compression technique using cuboid magnets (**A,B,C**).

**Figure 2:**
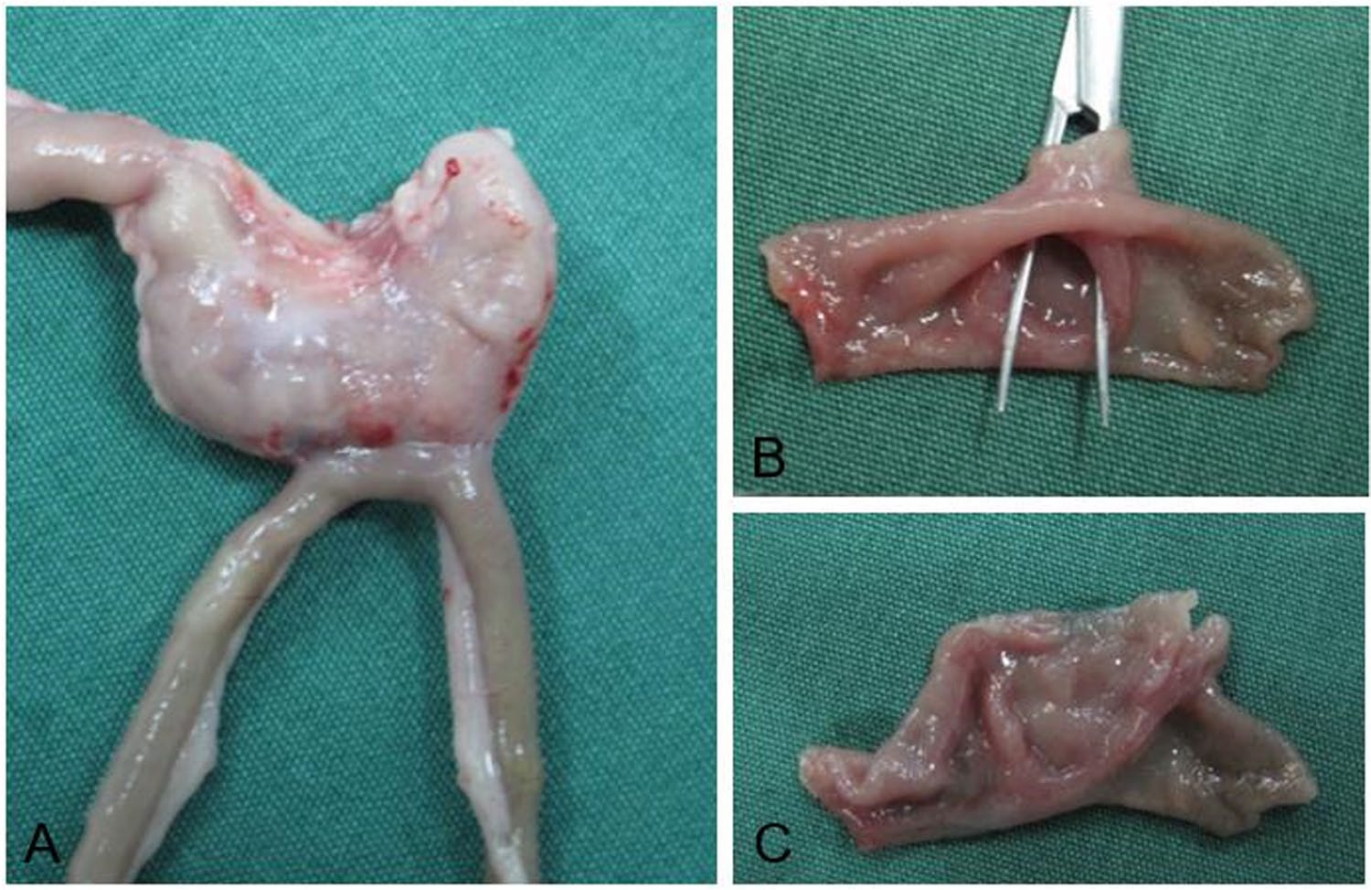
Gross appearance of anastomosis created by magnetic compression technique using magnetic rings (**A,B,C,D**).

The mucosa at the site of the anastomosis was grossly smooth and flat in both MCT-A (Fig. 1) and MCT-B anastomoses (Fig. 2). The mean anastomotic diameter was 5.07 ± 0.11 mm, 6.05 ± 0.08 mm and 7.06 ± 1.35 mm in MCT-A, MCT-B and hand-sewn group, respectively. The expected size, actual size and the rate of change are shown in Table 1.

**Table 1:** The size of gastrojejunostomy in each group.

	Expected size	Actual size	The rate of change, %
MCT-A	Cuboid,	D = 5.07 ± 0.11 mm,	
n = 10	S^a^ = 20.88 mm^2^	S^b^ = 20.17 ± 0.91 mm^2^	−3.40^c^
MCT-B	Ring,	D = 6.05 ± 0.08 mm,	
n = 9	S^d^ = 28.26 mm^2^	S^e^ = 28.74 ± 0.78 mm^2^	+1.70^f^
Hand-sewn	D = 6.50,	D = 7.06 ± 1.35 mm,	
n = 7	S^g^ = 33.17 mm^2^	S^h^ = 40.38 ± 14.42 mm^2^	+21.7^i^

^a^MCT-A: The length, width and height of the cuboid magnets was 7.20 mm, 2.90 mm, 1.60 mm, respectively. The anastomosis size of expected was 20.88 mm^2^;

^b^MCT-A: The diameter of anastomotic was 5.07 ± 0.11 mm, so the actual anastomosis size was 20.17 ± 0.91 mm^2^;

^c^MCT-A: The rate of change = S^b^–S^a^/S^a^;

^d^MCT-B: The outside diameter of the magnetic ring was 6 mm, so the anastomosis size of expected was 28.26 mm^2^;

^e^MCT-B: The diameter of anastomotic was 6.05 ± 0.08 mm, so the actual anastomosis size was 28.74 ± 0.78 mm^2^;

^f^MCT-B: The rate of change = S^e^–S^d^/S^d^;

^g^Hand-sewn: The diameter of rats’ jejunum was approximately 6.5 mm, and the cross-sectional area of the jejunum is taken as the expected size of the anastomosis. The anastomosis size of expected was 33.17 mm^2^;

^h^Hand-sewn: The diameter of anastomotic was 7.06 ± 1.35 mm, so the actual anastomosis size was 40.38 ± 14.42 mm^2^;

^i^The rate of change = S^h^–S^g^/S^g^.

### Histological appearance of anastomosis

**Figure 3:**
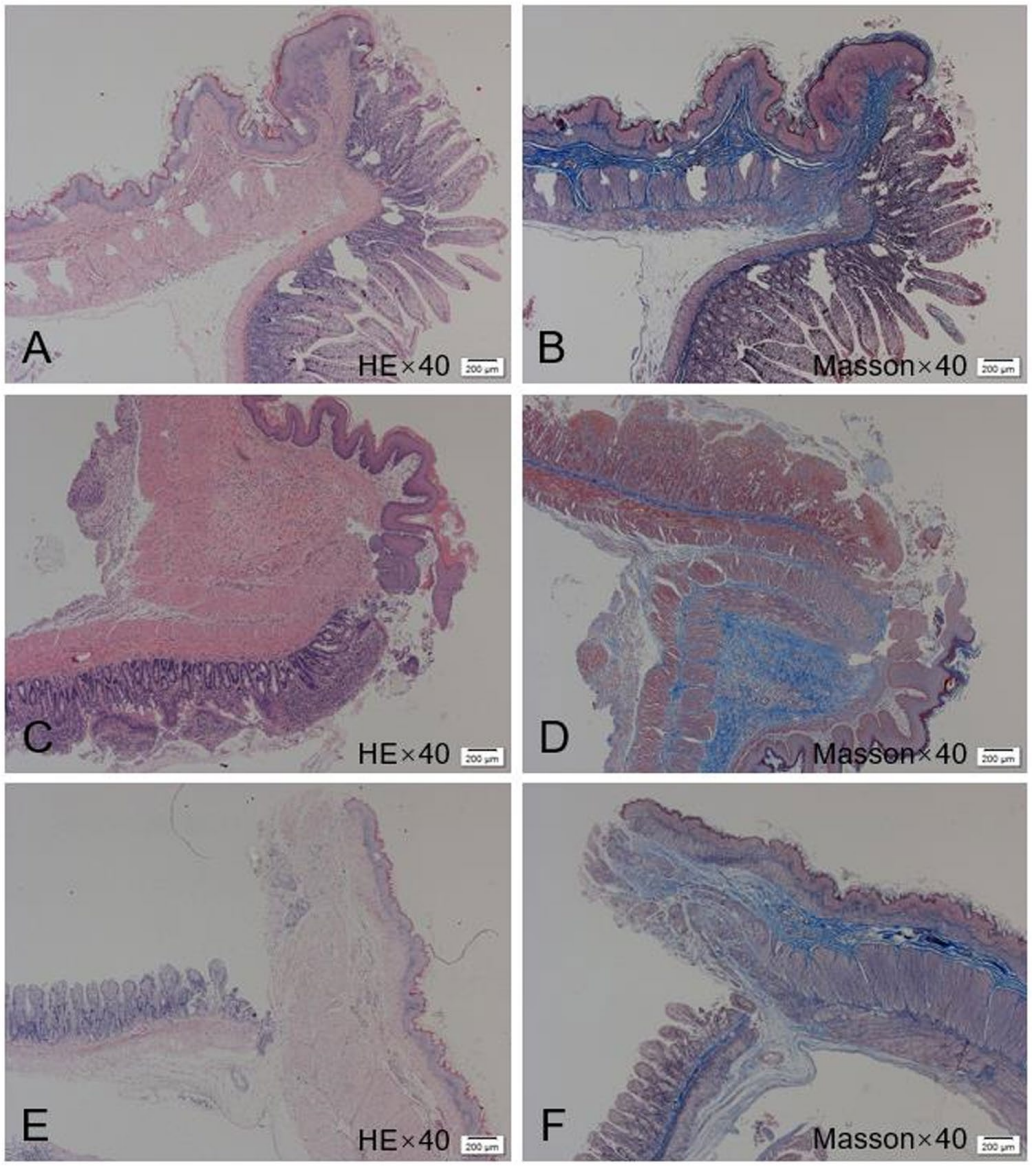
Representative histologic sections of the anastomotic sites of the study and control groups, one month after surgery (H&E & Masson’s, 40x). (**A**,**B**) MCT-A group: the mucosal layer was continuous with a relatively smooth surface and less collagen content in the muscular layer. (**C,D**) MCT-B group: Mucosal, submucosal and muscular with good continuity. (**E,F**) Hand-sewn group: mucosal continuity was slightly worse, the mucosal layer was continuous with presence of silk-related foreign body granulomas and mild inflammatory infiltration in the muscular and serous layer along with dense collagen deposition.

Histological examination of the anastomoses revealed the mucosal layer to be continuous with a relatively smooth surface in the MCT-A and MCT-B groups. Silk-related foreign body granulomas were absent and mild infiltration of inflammatory cells was observed in the muscular and serous layer. Four weeks after surgery, the anastomotic site in the study group had completely healed and the serosal, submucosal, and mucosal layers were continuous without ischemia or necrosis (Fig. 3A–D). In the hand-sewn group, mucosal continuity was slightly worse, the mucosal layer was continuous with the presence of silk-related foreign body granulomas and mild inflammatory infiltration in the muscular and serous layer along with dense collagen deposition.

## Discussion

Currently, hand-sewing and stapling are the most commonly used techniques for construction of gastrointestinal anastomosis in humans and other large animals such as dogs and pigs. However, dogs and pigs are not ideal animal models for metabolic diseases such as obesity and diabetes. The rat is a better model due to its small size, rapid breeding, and cost effectiveness^4^. However, hand-sewing intestinal anastomosis in small animals such as rats is technically difficult, time consuming and associated with high postoperative complications^20^. At present, no device has been validated for gastrointestinal anastomosis in rats.

MCT is a simple and feasible technique for gastrointestinal anastomosis. However, the gastrointestinal tract in rats is relatively small and use of magnetic compression technology for gastrointestinal anastomosis in rats has not previously been reported. Conventional magnetic compression anastomosis technology cannot be extrapolated in rats. Due to the different requirements for gastrojejunal anastomosis in rats, we designed two types of magnets.

In some studies, gastrojejunal anastomosis only provides a potential bypass, and the original digestive tract structure need not to be changed. Therefore, for such cases we adopted a novel method of oral magnet placement to achieve gastrojejunal anastomosis. This method does not require incision of the digestive tract, the magnets can be inserted through the mouth into the jejunum and stomach. The two cuboid magnets are attracted to one another and compress the wall of the stomach and jejunum causing formation of gastrojejunal bypass. Although this method cannot immediately establish gastrointestinal bypass, surgical trauma is minimized and the technique is very simple. All rats in this experimental group (MCT-A) survived. This method of gastrojejunal anastomosis establishment by implanting magnets through the oral route may be suitable for the clinical treatment of patients with patent gastric outlet.

In cases of gastric outlet obstruction requiring gastrojejunostomy, the magnetic rings can be used as illustrated in the MCT-B group. In this experiment, a central tube in the central hole of the parent magnetic ring has two important roles: (1), The central tube of the parent magnet ring provides access for the passage of chyme. However, the central tube of the magnetic ring should be large enough to avoid obstruction. In the MCT-B group, one case of postoperative obstruction occurred due to the blockage of the central tube by rat feed residue. (2), The center tube of the parent magnet ring facilitates appropriate coupling with the daughter magnet ring.

The size of gastrojejunostomy can influence the long-term postoperative outcomes. In this experiment, the diameter of the anastomosis at the time of surgery was smallest in MCT-A, followed by MCT-B, and maximum in hand-sewn group. However, simply discussing the size of the anastomosis can be misleading. In the hand-sewn anastomosis, the size of the anastomosis depends on how wide the surgeon constructs the anastomosis. On the other hand, in the MCT group, the size of the anastomosis depends upon the diameter of the magnets. So, an important aspect to be studied is the difference between the actual anastomosis size at autopsy and the expected size of the anastomosis at the time of surgery. According to Table 1, it can be seen that the actual size of the anastomosis in the MCT groups was most consistent with the expected size, and the standard deviation was small, suggesting that the stability of the anastomosis was better. While in the hand-sewn anastomosis group the actual size at autopsy exceeded the expected size with high standard deviation. This finding suggested that the size of the hand sewn anastomosis is less predictable compared to MCT due to varying degrees of inflammation.

This study shows that MCT can significantly shorten the gastrojejunal anastomosis time and simplify construction of anastomoses. Although the incidence of anastomotic leakage and obstruction in the MCT group was lower than that in the control group, due to the small sample size, this difference was not significant.

## Methods

### Ethical statement

This study was carried out in strict accordance with the recommendations of the Xi’an Jiaotong University Medical Center Guide for the Care and Use of Laboratory Animals. The protocol was approved by the Committee for Ethics of Animal Experiments at Xi’an Jiaotong University (Permit Number: 2018-001).

### Animals

Thirty male Sprague-Dawley rats (250–300 g) were obtained from the Experimental Animal Center, College of Medicine, Xi’an Jiaotong University (Xi’an, China). The animal protocol was designed to minimize pain or discomfort to the animals. The animals were acclimatized to laboratory conditions (23 °C, 12 h/12 h light/dark, 50% humidity, *ad libitum* access to food and water) for one week prior to commencing the experiments.

The rats were randomly divided into three groups: magnetic compression technique A group in which the anastomosis was performed using cuboid magnets (MCT-A, n = 10), magnetic compression technique B group in which the anastomosis was performed using magnetic rings (MCT-B, n = 10) and a control group in which hand-sewn gastrojejunal anastomosis was performed (n = 10). At the end of the study, all animals were euthanized by barbiturate overdose (intravenous injection, 150 mg/kg pentobarbital sodium) and tissues were collected.

### Magnetic Anastomosis device

The magnetic anastomosis device consists of two neodymium-iron-boron (NdFeB, N45) permanent magnets (parent and daughter) with a nitride layer coating to resist erosion (Northwest Institute for Nonferrous Metal Research, Xi’an China).

**Figure 4:**
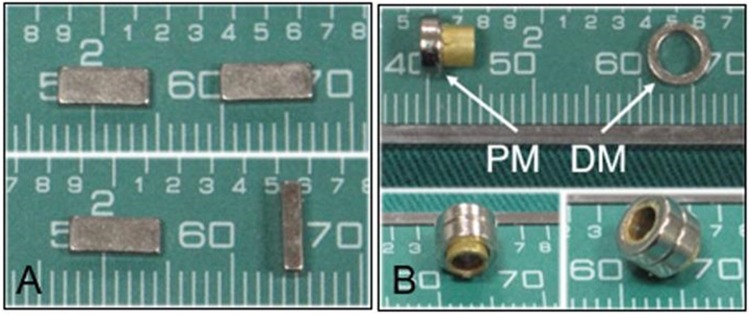
The magnetic device (**A**), cuboid magnets (**B**), parent magnet (PM) ring and daughter magnet (DM) ring.

Two different types of magnets were designed to meet surgical requirements. The first design consisted of a pair of cuboid magnets of the same size and shape (Fig. 4A). The length, width and height of each magnet was 7.20 mm, 2.90 mm and 1.60 mm, respectively, and the weight was 0.25 g. The second design included parent and daughter magnetic rings (Fig. 4B), with an outer diameter of 6 mm, inner diameter of 4 mm and thickness of 2 mm. The inner diameter of the parent ring central tube was 3 mm. The parent and daughter magnet rings were 0.44 g and 0.23 g, respectively.

### Study procedures

The rats were weighed and anesthetized by intraperitoneal injection of chloral hydrate (350 mg/kg) after one day of fasting. After loss of the paw withdrawal reflex was confirmed, the animals were fixed to a temperature-controlled operating table and their abdomen was shaved. Sterile surgical instruments were used for surgical procedures. In all experiments the abdominal cavity was accessed with a 3-cm craniocaudal midline incision. The stomach and jejunum were then identified, moved out of the peritoneal cavity and placed on a sterile gauze and hydrated with sterile saline solution to prevent dehydration. Gastrojejunal anastomosis was then performed.

In the control group (conventional hand-sewing method) a loop of the jejunum about 10 cm distal to the Treitz ligament was isolated and side-to-side gastrojejunal anastomosis was constructed with 6-0 absorbable sutures (Ethicon, Johnson & Johnson). About 0.5 cm distal to the pylorus, the duodenum was doubly ligated using 1-0 non-absorbable silk suture.

**Figure 5:**
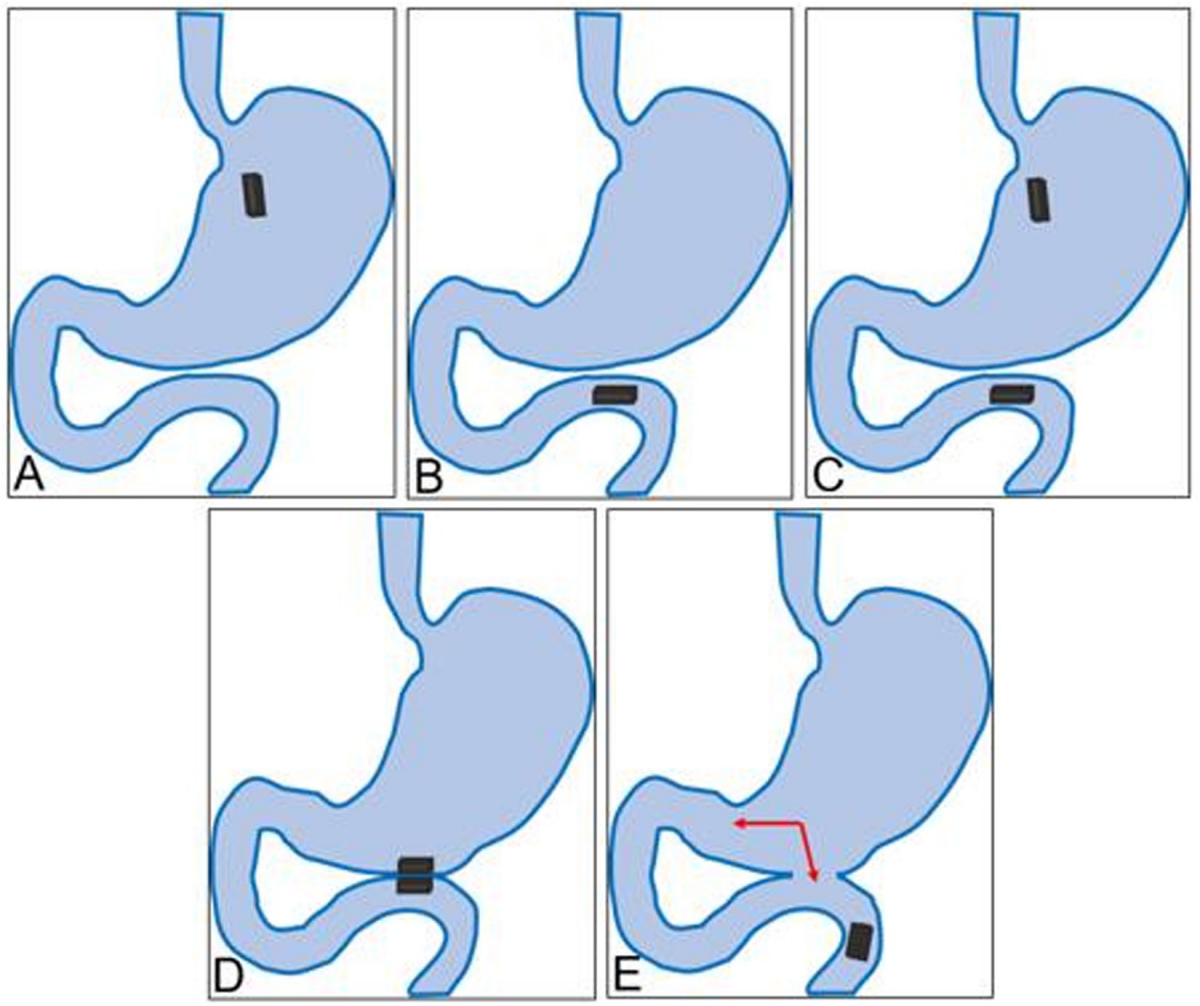
The MCT-A group surgical procedure. (**A**) The magnet is introduced through the mouth into the stomach; (**B**) The magnet is further pushed into the jejunum; (**C**) The second magnet is pushed into the stomach through the mouth; (**D**) The two magnets are attracted to one another; E: 5–10 days after surgery, the magnets are expelled from the rat.

**Figure 6:**
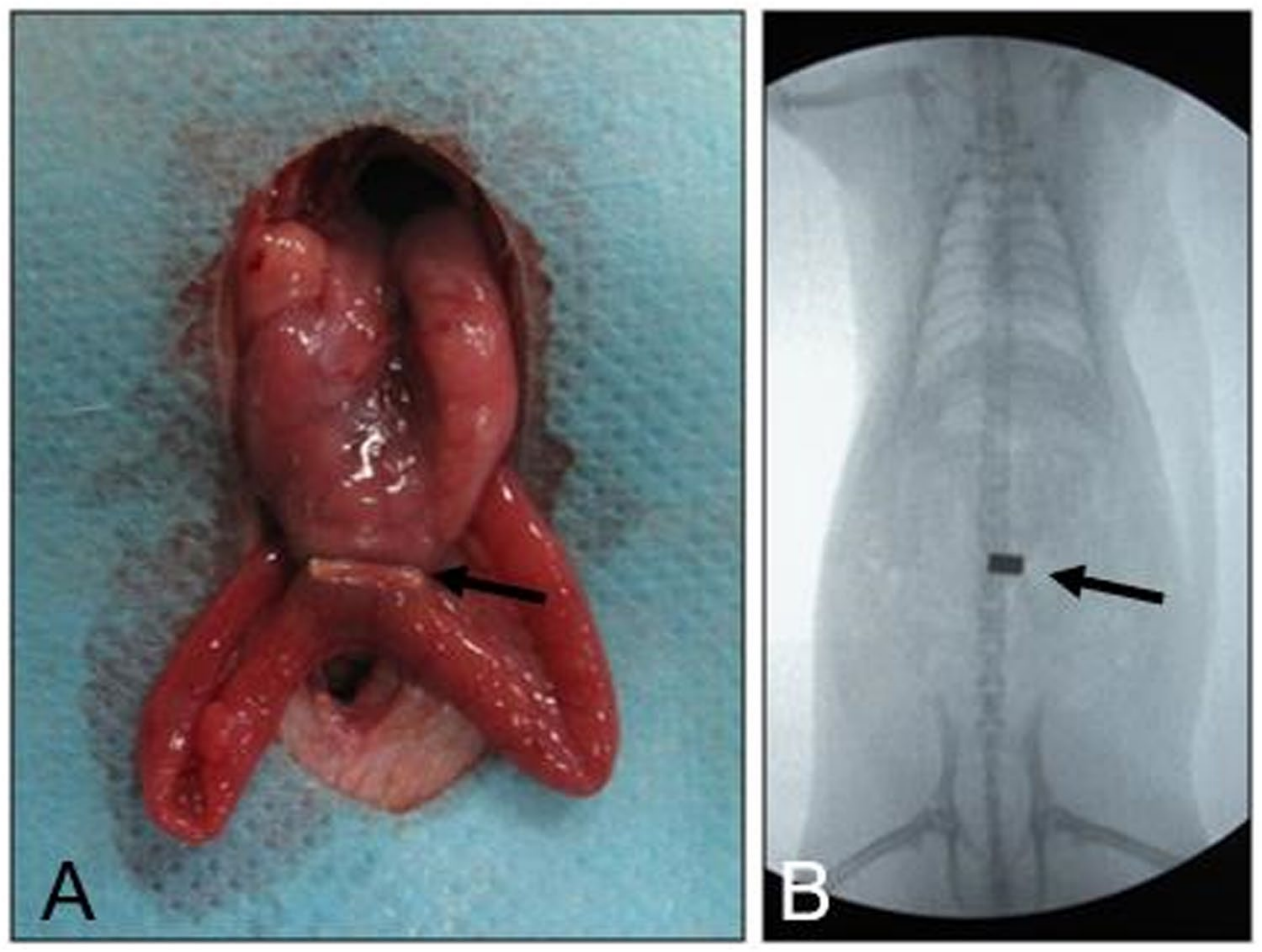
(**A**) The two cuboid magnets are attracted to one another; (**B**) Abdominal fluoroscopy shows the position of the magnets.

In the MCT-A group, the following surgical procedure was followed: the magnet was placed into the stomach through the mouth (Fig. 5A). The magnet was pushed into the jejunum manually without fluoroscopic guidance. (Fig. 5B). Using the same method, the second magnet was placed into the stomach (Fig. 5C). The two magnets were positioned in close proximity, resulting in mutual attraction which compresses the intervening stomach wall and jejunal wall, leading to the construction of gastrojejunal anastomosis (Figs 5D, 6A).

**Figure 7:**
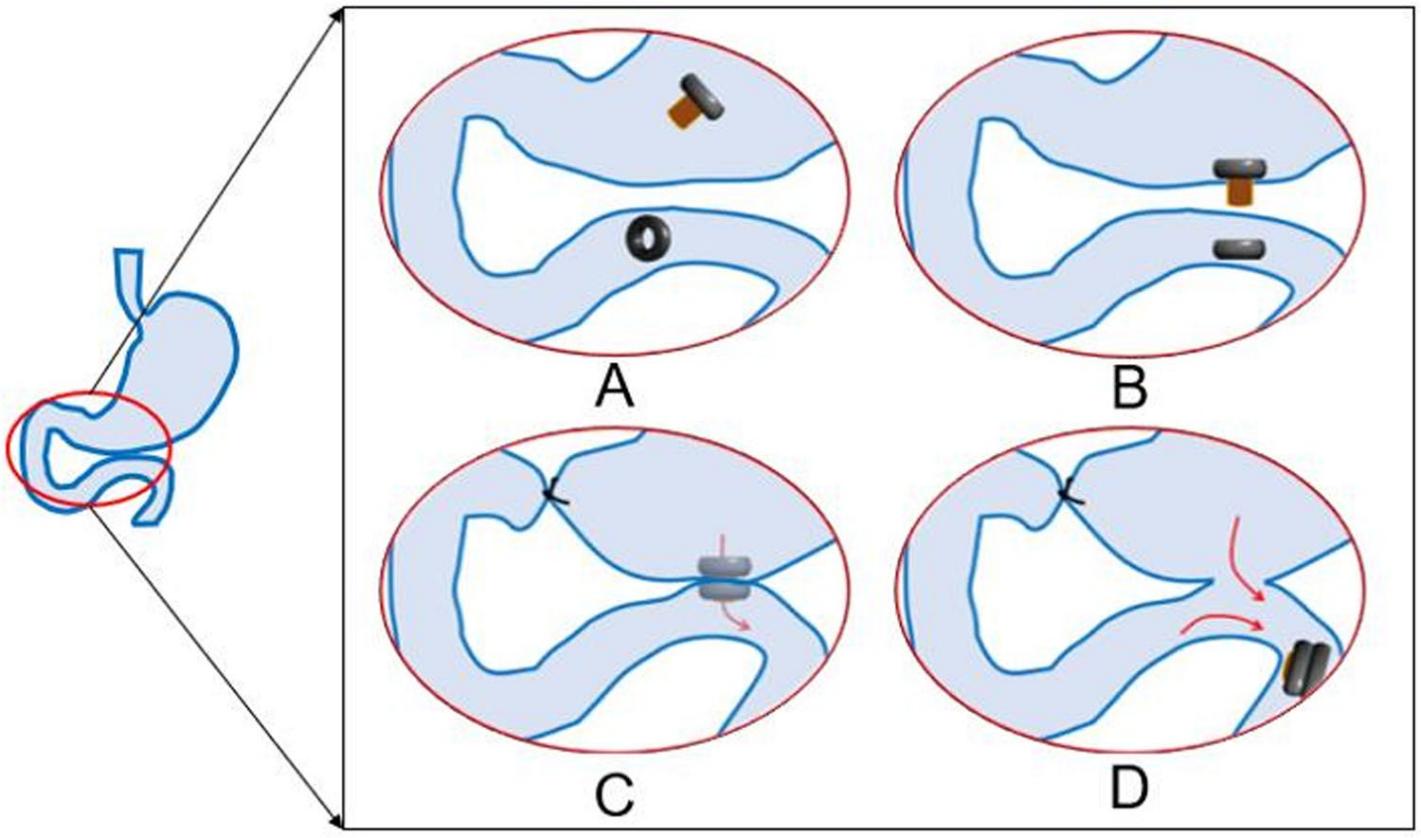
The magnetic ring surgical procedure. (**A**) The daughter magnetic ring is placed into the jejunum, and the parent magnetic ring into the stomach through an incision in the stomach wall; (**B**) The parent magnetic ring is adjusted such that the central tube of the magnetic ring projects through the stomach wall; (**C**) The parent and daughter magnetic rings are attracted to one another; (**D**) 7–12 days after surgery, the parent and daughter magnetic rings are expelled.

**Figure 8:**
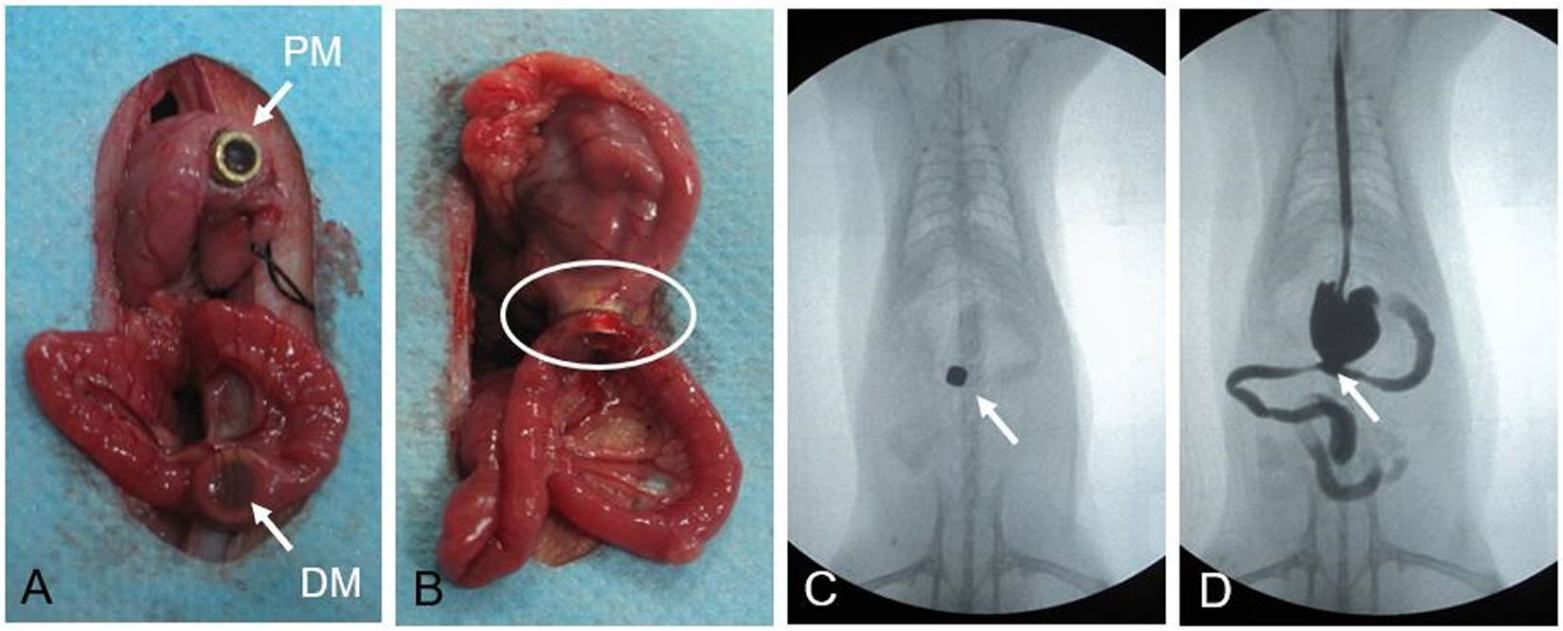
(**A**) The parent magnetic ring and the daughter magnetic ring; (**B**) The parent and daughter magnetic ring are attracted to one another; (**C**) Abdominal fluoroscopy shows the position of the magnetic rings; (**D**) Upper gastrointestinal contrast radiography showed anastomotic patency.

In the MCT-B group, the following procedure was followed: a purse - string suture was taken in the body of the stomach using 4-0 non-absorbable silk. A 4-mm incision was made in the center of the purse suture, the daughter magnetic ring was inserted through this incision and pushed into the jejunum manually without fluoroscopic guidance. The parent magnetic ring was inserted in to the stomach through the same incision (Figs 7A, 8A). The parent magnetic ring was placed such that the central tube of this magnetic ring projected through the stomach wall (Fig. 7B). The two magnetic rings were brought close to each other resulting in mutual attraction. At the same time, the central tube of the parent magnetic ring cuts the jejunum wall covering the daughter magnetic ring. The intestinal contents could pass through the central tube (Figs 7C, 8B). About 0.5 cm distal to the pylorus, the duodenum was doubly ligated using 1-0 non-absorbable silk suture.

### Calculation of anastomosis time

The anastomosis time was recorded from the beginning of anastomosis construction to its completion. The anastomosis time was recorded for each rat.

### Postoperative care

Immediately after surgery, a plain abdominal X-ray was taken to confirm accurate coupling of the magnetic compression rings (Figs 6B, 8C). In the MCT-B group, rats underwent contrast examination to confirm patency of the alimentary tract (Fig. 8D). After recovery from anesthesia, the animals were allowed a liquid diet and water. During the first seven postsurgical days, the rats were fed with liquid, and thereafter normal feeding was given.

### Bursting pressure

Four weeks after surgery, the rats were euthanized with high dose barbiturate. A 10-cm anastomosis-bearing segment of the stomach and jejunum was resected with the adherent organs and one end of the anastomotic specimen was clamped by hemostatic forceps. A catheter was introduced through the other end and ligated with a single silk suture. The whole anastomosis was immersed in 0.9%NaCl. The bursting pressure was measured by a pressure gauge. The intraluminal pressure was then gradually increased, and the point at which the first air bubble rose to the surface of the liquid was recorded. The intraluminal pressure at which the air leakage started was recorded as the bursting pressure.

### Histological analyses

A sufficient length of the anastomosis-bearing segment of the stomach and jejunum was cut and immersed in 10% buffered formalin overnight. After fixation, the anastomosis-bearing segment was embedded in paraffin, and 4-µm thick sections were cut at the site of the anastomosis. Sections were stained with hematoxylin and eosin (H&E) or Masson’s trichrome stain and examined under a bright-field microscope.

### Statistical analysis

SPSS statistics 17.0 software was used for data analyses. Quantitative data are expressed as mean ± standard deviation. A P value < 0.05 was considered to indicate statistical significance.

### Data availability

No datasets were generated or analyzed during the current study.
